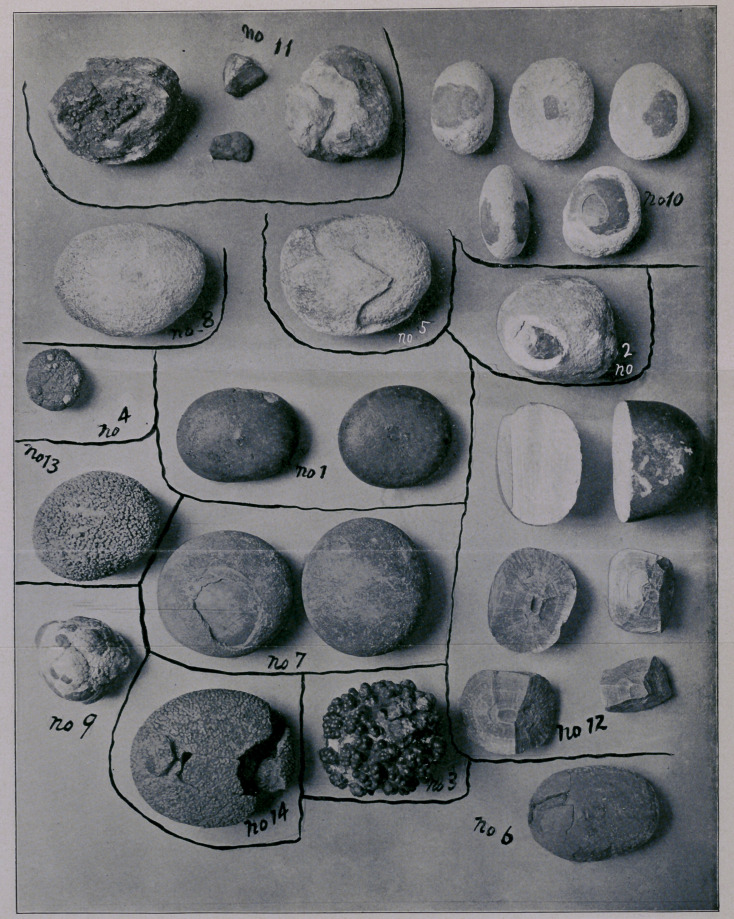# Stone in the Bladder and Lithotomy*Read before the Texas State Medical Association, Waco Texas, April 26, 1900.

**Published:** 1900-06

**Authors:** F. Paschal

**Affiliations:** San Antonio, Texas


### TEXAS MEDICAL JOURNAL.

AUSTIN, TEXAS.

ESTABLISHED JULY, 1885.

PUBLISHED MONTHLY.—SUBSCRIPTION $1.00 A YEAR.

VOL. XV. AUSTIN, JUNE, 1900. No. 12.

### Original Contributions.

For Texas Medical Journal.

### Stone in the Bladder and Lithotomy.*

BY F. PASCHAL, SAN ANTONIO, TEXAS.

The history of operations for stone in the bladder dates back over two thousand years, and then our ancestors in surgery tried to find the best route to the bladder, and we are today following in their footsteps. Just as they accepted one method of operating and discarded it, so are we seemingly doing.

But before comparing the merits of the different operations, it may not be unprofitable for us to go briefly into the causes of the formation of stones, for after all this is the most important part bearing upon its treatment, for we know full well that in removing a stone from the bladder we simply rid the patient of a local condition which is apt to recur unless intelligent treatment is employed to prevent its formation.

In the first place, stones have for their bases organic and inorganic substances; those having the organic base are from the kidneys, are the most frequent, and are classed as uric acid, urates, and their modifications, the oxalates, uric oxide and cystic oxide. The stones having an inorganic base are composed principally of phosphates of lime, magnesia, and ammonia, and those rare varieties, the carbonate of lime and the fibrinous and uro-stelath, and are usually caused by local bladder trouble. The primary cause of the formation of the

*Read before the Texas State Medical Association, Waco, Texas, April 26, 1900.

stones having the organic base is supposed to be due to the so-called improper combustion and improper assimilation of food, which produces an over elimination of normal substances found in the urine. The exact vice of constitution that gives rise to this over-production or chemically changed substances, usually eliminated in a dissolved state, is considered to be diathetic, acquired or hereditary. According to Murcheson, functional derangement of the liver is one of the principal causes for the so-called improper combustion of food, but the fact remains that food and mode of living have a direct bearing upon the cause of the formation of organic stones.

In statistics collected by Civeale, Coulson, and Thompson, of 10,-467 cases, 62.33 per cent. were under twenty years of age, and almost all of them were derived from hospital practice; but Sir Henry Thompson found that this percentage in his private practice was not so large, and accounted for it by the neglect and indifference to symptoms in the average of the poorer class, and also to bad hygienic surroundings, and from mal-nutrition and irregular diet of children in the lower walks of life. The experience of others with stone is in accord with Sir Henry Thompson's statement. The operations that I have done have been upon the poorer classes, and upon children badly nourished and subjected to unhygienic surroundings. It has fallen to my lot to do forty-three lithotomies, and thirty-nine of this number have been upon Mexican subjects, and all of them chiefly amongst the poorer class. Of the forty-three cases, nine were from two and one-half to twenty years of age, and thirty-four were adults from twenty to eighty years.

A direct relation can also be traced between the frequency of stone amongst the poorer classes of Mexican people, as well as those of other races, for in the upper walks of life, owing to better care and food, stone is not frequent. The food upon which the poorer class of Mexicans live is almost entirely hydrocarbonaceous, and consists of corn, beans and sugar, and occasionally a little dried beef. Their surroundings are unhealthy, and manual labor, aided by the heat-forming diet, produces free perspiration, and this, combined with the improper assimilation of food, adds an increased amount of organic salts, which, instead of passing out of the kidneys in a dissolved state, becomes crystallized and precipitated in the kidneys or bladder, and there begins the formation of stone in the bladder. Sometimes these crystals in the kidneys are so large that in passing down the ureters they cause intense pain or that never-to-be-forgotten agony commonly known as a "fit of the gravel." If, as more frequently happens, these crystals pass out of the bladder, no local harm is done, and aside from the multitudinous and complex symptoms and diseases that this stone-forming condition gives rise to nothing is noted; but, if the almost microscopic crystal lodges in the bladder and remains there, a catarrhal condition is soon developed and an albuminoid or colloid substance is secreted by the mucous coats of the bladder which gather up and seal the crystallized salts in the urine and form them together in a stone in the bladder, varying in weight from a few grains to huge boulders, weighing as much as six pounds, three ounces, and in numbers from a single stone to one thousand.

It can be readily seen from the history of the mode of life amongst the Mexicans that the same underlying cause will be found in most instances to exist in other races, notably those of India, and, as stated before, the treatment of the cause that gives rise to the formation of the stone is of far more importance than the mere mechanism of the operation for removing the stone. I would not, however, insult the dignity of this body by trying to map out how these cases should be treated, for each will be found a case unto itself, and what might be applied to one could not be employed in another, for surroundings, and means to procure proper food and better care do not always go together, so that all we can do is to tell them how they ought to live, and we may grow gray and not succeed in teaching people this, because they will not follow our advice on this particular point; they will take medicine with an avidity, but when it comes down to restraining them from eating what is harmful, and living as they should live, they will simply not obey our instructions.

The causes of inorganic stones are usually local and in the bladder; hence the cause must be sought and removed.

There are two methods of performing lithotomy—the suprapubic and the perineal. The perineal operation is the most ancient and was practiced upward of two thousand years ago. It was known as cutting on "the gripe," and was done by introducing two fingers into the rectum, gripping the stone and pressing it against the perineum, where it was cut open and removed. It was really the operation ot Celsus, or the operation of the apparatus minor.

The lateral operation remains today pretty much the same as it was in 1697, when Jacques did the operation with a sound, having cut, it is said, five thousand times; but the credit of perfecting the operation is usually awarded to Cheselden.

The suprapubic operation was practiced in Paris, in 1485, by Colát, as an experiment on a criminal by permission of Louis XI, and the patient recovered. The earliest account of this method is published in 1556, by Pierre Franco. He performed it on a child three years old after finding the calculus too large to be removed through the perineum. His remarks then tended to discourage the practice. There seems to be a tendency today to abandon the perineal operation for stone and give preference to the suprapubic method; and it is in defense of the perineal operation that I have written this paper.

In looking into the past history of the operations, I find that Jacques, Como, Douglas, Cheselden, Paul, Pye, Molgil, Thombill, and Suberville abandoned the suprapubic operation on account of the large mortality, which amounted to one death for every 4.08⅓ cases cut, and accepting the perineal, which gave a mortality of one in every eight cases operated upon. The result obtained by these operators could not have been due to inferior methods of operating, for since the introduction of antiseptics, or the aseptic era, the mortality is still in favor of the perineal method; but just to what extent, it is impossible to say definitely, as tables of different authors vary.

Statistics since 1878, given by Morrow, give a death rate in children under fourteen years of age of 12.52 by the suprapubic operation; and by the perineal method, a death rate of 2.96; adults from fourteen to fifty, by suprapubic method, 12; by perineal method, 11.36; old men, over fifty years of age, 32.07 by suprapubic, and 16.66 by perineal method.

According to Cabot, of 226 perineal lithotomies, from puberty to middle age, there were twenty-two deaths, or 9.7 per cent. mortality. Suprapubic lithotomy, 159 cases, eighteen deaths, or 11.3 per cent. mortality; litholapaxy, 485 cases, twenty-two deaths, or 4.5 per cent. mortality. In old age, perineal lithotomy, sixty-nine cases, thirteen deaths, or 19 per cent. mortality. In old age, suprapubic lithotomy, ninety-one cases, seventeen deaths, or 18 per cent. mortality. Litholapaxy, 581 cases, forty deaths, or 7 per cent.

It will be seen that from puberty to middle age the mortality was 1.6 per cent. in favor of the perineal operation; and in old age one per cent. in favor of the suprapubic operation. The analysis of Morrow's tables gives the following:

In children under fourteen years of age there is a difference in mortality of 9.56 per cent. in favor of the perineal operation. In adults from fourteen to fifty years of age, .64 of one per cent. in favor of the perineal operation. Old men over fifty years of age, 14.41 per cent. in favor of the perineal operation. The percentage from fourteen to fifty years of age in both operations is about the same, but under and above these ages, the death rate is considerably in favor of the perineal operation.

In Thompson's tables, dating from 1790 to 1840, of 1287 perineal lithotomies of all ages, there were 229 deaths, or one in every eight cases operated on, which equals 12.5 per cent. mortality.

Barling's tables, from 1888 to 1892, of 613 cases of all ages, there were sixty-one deaths, or one in every ten cases operated on, which is equal to ten per cent. mortality.

The following significant extract is taken from Dennis' Surgery: "These figures emphasize the fact that the general mortality of stone operations is reduced, but it appears to show also unmistakably that operations for stone in children are attended with greater mortality than they were fifty years ago. Barling says: 'This, if it be true, can only be due to the introduction of litholapaxy and suprapubic section, and to the latter the enhanced mortality must be ascribed.' He further says: 'Lateral lithotomy and litholapaxy are safer operations in children than the suprapubic is at present, and that if the last mentioned is adopted as a routine procedure, it must be shown to give better results than it does now.' "

It is difficult, if not impossible, to be guided by statistics, as operators do not always publish their failures, but never neglect to record their successes when occasion requires. The choice of an operation must be largely a matter of personal experience, and the surgeon who adheres to one operation and perfects himself in it will be more apt to have better results than the one who follows fashion for fashion's sake and first tries one and then the other operation. That litholapaxy gives the lowest death rate seems from comparison evident, but there are few general surgeons who are fortunate enough to have a sufficient number of stone cases to make them familiar with this operation. Many physicians may go through years of active practice and never meet with a single case. Hence it is safer for the surgeon, unless he is skilled in litholapaxy, to do a perineal or suprapubic lithotomy in preference to litholapaxy.

Keegan says: "The surgeon who meets with cases of stone only at rare intervals during his career will be acting more wisely if he adheres to lateral lithotomy or suprapubic cystotomy. It is his misfortune and not his fault, that he has not been afforded many opportunities of gaining a practical familiarity with the use of the lithrotrite."

P. J. Freyer has performed 912 operations for stone in the bladder. Of these 249 were perineal and seven suprapubic, showing that when he does the cutting operation he prefers the perineal.

Keyes says: "If the cutting operation is to be performed, the suprapubic route is to be chosen, as this permits of more perfect work and allows the surgeon to remove obstructions. The only safe guide is surgical judgment based upon diagnosis."

Cunningham, in presenting the records of 123 operations, says that not over three per cent. are unsuited for litholapaxy. If compelled to cut, he greatly prefers the perineal route to the suprapubic.

In comparing the death rate for cases of all ages, cut in olden time, and to the present, it is, according to those found to be recorded, in favor of the perineal operation.

Some of the reasons advanced in favor of the suprapubic operations are that it traverses no important structures and leaves no bad consequences; but this is not always so, for the peritoneum is sometimes wounded, infiltration of urine occurs and fatal peritonitis sometimes follows, and fistulæ are not infrequent. In the past six months I have seen two large ventral herniæ following the operation of suprapubic cystotomy.

Against the perineal operation it is said that it traverses more important structures to reach the neck of the bladder; that wounding of the ducts may bring on impotence, and that fistulæ are more frequent, and that the rectum is sometimes wounded.

The answer to these objections is that the danger of impotence is hardly worthy of serious consideration. In the lateral operation the prostate is divided on the left side, and as the duct penetrates the lower part of the substance of the gland in order to reach the urethra, and as the knife divides the side of the gland obliquely inwards and upwards, or outwards and downwards, the duct will not be in danger; and even if it should be cut, the duct on the right side will remain uninjured.

If the rectum is wounded low down it is of no great importance, and with ordinary care and skill, it should not be wounded high up. It is claimed that there is danger from incontinence of urine following this operation, but this is so slight as hardly to be considered.

Drainage, it is claimed, is more perfect after the suprapubic. How can it be? You drain against gravity in the suprapubic operation, and by gravity in the perineal. Then, again, what is the use of drainage in the perineal operation? For only in very exceptional cases can there be any need for it. That there are cases in which the suprapubic method is preferable cannot be denied; but taking the ordinary run of cases, it will be found that the perineal operation will give equal, if not more satisfactory results.

In summing up, it may be said:

*First.*—That each operation has its indications and must be chosen to meet the merits of the case.

*Second.*—That litholapaxy gives the lowest mortality, but very hard and large stones, and stones with hard, foreign bodies in them as a nucleus, are not suited for this operation.

*Third.*—That in all stone cases there is a chronic cystitis, and it is desirable to give the bladder rest; litholapaxy only does this by removing the stones, whereas there is no better treatment in many cases of cystitis than by opening the bladder and thereby giving it rest.

*Fourth.*—In unskilled hands litholapaxy is not so safe as the cutting operation.

*Fifth.*—The perineal gives a smaller death rate and the dangers attending it are no greater than in the suprapubic operation.

*Sixth.*—The suprapubic is best suited for cases of very large stones, in encysted stones, and in old men and with prostatic tumors or enlargements. It is unsuited for children.

*Seventh.*—A well-performed lateral operation leaves little to be desired; is no more difficult of execution, and is suited to cases of all ages.

*Eighth.*—The combined suprapubic and perineal operations may be done with perfect safety, as in Case 43 herewith reported.

*Ninth.*—Perineal lithotrity is not a very well accepted operation. It is frequently done, but it has no particular advantages over the other operations.

*Tenth.*—The recurrence of stone in the bladder after its removal sometimes follows all the different operations, and, perhaps, with no greater frequency in one than in another of the methods employed for its removal. They more frequently recur after the age of forty. In old men, in chronic cystitis, with enlarged prostates, and in saculated bladders. Hence the cause that gives rise to the formation of stones is of the first importance and should be sought.

Histories of stone cases are usually of the same character. But I will give a few cases, as they are exceptional in some respects.

Case No. 24, age 62; operated June 23, 1886; lateral lithotomy. Removed two stones, each weighing 169 grains. From color of stones, probably uric. In removing the stones, a tumor of the prostate, about the size of an almond, was removed. No bad results followed. Acute orchitis developed fifteen days after the operation.

Made a rapid recovery from it. August 17, 1892, seen today; health excellent.

Case No. 18 was that of a celebrated thief known as the "Peacock." He was thirty years old, about six feet one inch, and built proportionately; a splendid specimen of a physical man, but a very devil. In 1878, while he was trying to make his escape after arrest, he received a gunshot wound from a forty-four caliber pistol fired by a policeman, the distance between the officer and thief not being more than three feet. I saw him in half an hour after he received the wound, and found that the bullet had entered about two and one-half or three inches above the pubes and slightly to the right of the median line. There was some pain, but no shock. He had no escape of urine through the wound. He had retention of urine for twenty-four hours. The bladder could be plainly seen well distended; still there was no leakage through the external wound. Shortly after twenty-four hours he passed water through the urethra, somewhat bloody, but without pain. He had circumscribed area of tenderness over lower part of the abdomen, but nothing of importance. There was never a rise of temperature. He remained in bed about ten days and I never saw him again for five years, when he consulted me with all the symptoms of stone in the bladder. I examined and found a foreign body in the bladder, and expressed my belief that it was the bullet, and proposed an operation. He refused, and once more went to stealing and robbing. Two years afterwards, or seven years from the time he was wounded, he called on me again, his sufferings aggravated. I again proposed the operation, but before consenting he was arrested, put in jail, and subsequently made a soldier of. After serving as a soldier for six months, he sent me word that if I would get him out of the army he would let me cut him. I gladly complied with his request, being anxious to investigate my diagnosis. But instead of undergoing the operation, he skipped out of the hospital, and went to thieving again, and I never saw him for six years, or thirteen years from the time he was wounded, when he turned up in my office one day and said: "Now my sufferings are so great I will let you operate." I did the lateral operation, and told my assistants what I expected to find in the bladder. In removing the stone it was entirely encrusted with phosphates, and I saw a smile on their faces at the expense of my mistaken diagnosis; but slightly scraping the point of the stone plainly revealed the bullet as its nucleus. He made an uninterrupted recovery, but the knife that I cut him with was stolen from amongst my instruments that I left at his house to be cleaned.

Case No. 7, age 20; operated March 18, 1879; lateral lithotomy; oxalate calculus; weight, 450 grains. This poor fellow was blind, half-witted, and had this affliction. He recovered. The stone is a beautiful specimen of the mulberry variety.

Case No. 12, age 5; removed June 16, 1891; lateral lithotomy. Union by first intention. Weight of stone, 55 grains; oxalate.

Case No. 1, age 60; removed May 10, 1877; recovered. Following note made August 17, 1884: "Heard from today; is still well. His first wife died and he married again and had one child."

Case No. 8, age 56; removed May 30, 1879; lateral lithotomy. Union by first intention. Twenty-four hours after the operation urine began passing through the urethra; not a drop passed afterwards through the wound. The wound was entirely healed in seven days. Urate stone; weight, 200 grains.

Here, then, we have two cases giving primary union after perineal lithotomy, and I ask the gentlemen favoring the suprapubic operation to show any better results.

Case No. 43. A. Green, age 57; married; occupation, retired merchant. December, 1897, commenced having hæmaturia and had pain and difficulty in micturition. Consulted a physician, who prescribed for him and washed out the bladder and treated him for two or three weeks, and then took him to a specialist, who also examined and treated him, but with no relief. He was then advised to go to New Orleans, and August 28, 1898, found him there, where he was examined and calculus discovered. He was operated on between the 1st and 5th of September, 1898, but with no relief. He was told ten days after the operation that he would have to undergo another one, to which he consented, and which was performed, but also without relief. He became very weak, and at times delirious. He remained in the Infirmary for four months, and after getting out of bed could scarcely walk for three or four weeks. He stated that he complained a good deal to his physician, but was told that nothing more could be done for him.

He left the Infirmary on January 13, 1899, and arrived in San Antonio January 15, 1899, with the wound unhealed. His suffering continued, micturition being about every half hour. He was referred to me by Dr. W. L. Barker, of San Antonio, and was admitted into the City Hospital December 4, 1899.

Aside from his suffering, his general condition was good. Upon examination, I found that a suprapubic and also a perineal operation had been performed. Hernia had resulted from the suprapubic operation on account of tenesmus. The hernia was quite large and extended to the pubes. Sounding revealed the presence of a large stone or stones.

After due preparation, I operated on him December 8, 1899, first doing the lateral operation, because I thought the stone could be easily removed, and also on account of the hernia, and because of the preference for this operation. After entering the bladder, I found a large stone which was easily removed, and upon further examination, I felt a tumor in the right horn of the bladder, so to speak, which felt to be about the size of a goose egg. I could feel no open surface, but was satisfied that I had to deal with an encysted stone, and, therefore, lost no time in doing the suprapubic section. On making the incision, a loop of intestine dropped into the wound and it was pressed back into the abdomen and gauze placed to hold it out of the way. After reaching the bladder, two silk ligatures were placed in the bladder and the bladder opened between them. Upon introducing the finger, the tumor was found to be an encysted stone, and a small surface just about large enough to admit the tip of the finger was all of the uncovered surface of the stone. It was with some difficulty that the stone was peeled out of its bed and removed. The gauze packing was left in the abdomen two or three days and then removed and replaced, and in about seven days discontinued. Intestines thereafter gave no trouble.

The patient made an uneventful recovery, and left the hospital in five weeks with wounds healed and hernia much improved. Has continued well. I saw him on April 18, 1900, and he can go all night without urinating.

The points of interest in this case are: The tolerance of the bladder to surgical interference, it having been opened four times, twice by the perineal and twice by the suprapubic method; the absence of peritoneal disturbance following the bathing of the intestine in urine; also the extent to which suffering will drive one to seek relief. It is really hard to decide which to admire most—the tolerance of this patient to surgical interference, or his faith in doctors after passing through what he did.

This case exemplifies the necessity of careful examination in all bladder cases. I do not believe that one physician should be any more skilled than another in making out stone in the bladder, if ordinary care and pains are taken. And if there is a stone in the bladder it should always be found, even if it is necessary to do a suprapubic or perineal exploration. Small stones may escape detection on first examination, but one examination should not suffice, and the examinations ought to be repeated several times until thoroughly satisfied that there are no stones in the bladder. Large stones are detected without difficulty.

Exactly what was done in this case I do not know, as he got no relief after the first operations; it is, therefore, natural to infer that the source of his trouble was not removed.

In conclusion, I beg to exhibit these specimens, as they may interest some of you, and will say that if my experience is worth anything, I am willing to continue the lateral operation, having had two deaths in forty-three cases—and they were men, one of sixty and one eighty years old. It should also be remembered that these operations were done in the very worst surroundings imaginable, and often nothing but an undressed sheep skin served as a bed.

If the suprapubic operation can be proven to give better results than the lateral, I shall willingly accept it.

### EXPLANATION OF PLATE.

The photograph gives the actual size of the stones.
No. 1 refers to Case No. 24.
No. 2 refers to Case No. 18.
No. 3 refers to Case No. 7.
No. 4 refers to Case No. 12.
No. 5 refers to Case No. 1. Urates and phosphates; weight of stone, 420 grains.
No. 6 refers to Case No. 8.

No. 7.—Two urate stones from bladder of same person; combined weight, 840 grains. Age of patient, sixty-five years. Removed in 1890. No recurrence. Recovered.

No. 8.—Urate and phosphate stone; weight, 352 grains. Age of patient, twenty-five years. Removed in 1881. Recovered. No recurrence.

No. 9.—Oxalate stone; weight, 320 grains. Age of patient, twenty years. Removed in 1877. Recovered. No recurrence.

No. 10.—Five uric stones with phosphatic coverings. Combined weight, 552 grains. Age of patient, sixty-five years. Removed May, 1882. Recovered. No recurrence.

No. 11 refers to Case No. 43. Nuclei—the small pieces; composition of stones, uric and phosphatic. Combined weight, 830 grains.

No. 12.—Two uric stones. Combined weight, 1080 grains. Age of patient, sixty years. Removed 1879. Died two months after operation of chronic enteritis. Stratification shown and also where the nucleus lay. One stone fractured. The other sawn through to show formation.

No. 14.—Uric stone; weight, 400 grains. Age of patient seventy-four years. Removed 1882. Recovered. Reformed in two years. No secondary operation. Died from old age and exhaustion. Fractured by falling.

## Figures and Tables

**Figure f1:**